# The First Lines of the Theory and Practice of Surgery; Including the Principal Operations

**Published:** 1845-02

**Authors:** 


*The first lines of the Theory and Practice of Surgery; including the principal operations.* By Samuel Cooper, Senior Surgeon to University College Hospital, and Professor of Surgery in the same College, etc. *With notes and additions,* by Willard Parker, M. D., Professor of Surgery in the College of Physicians and Surgeons in the University of the State of New York, etc. etc. *Fourth American, from the Seventh London Edition. In two volumes 8vo.* New York: Samuel S. and William Wood. 1844.

The Surgical writings of Samuel Cooper, and particularly the present work, are so well known to the profession in this country, that nothing more seems necessary than to notice a new edition, under the supervision of a competent Editor.

"This work was originally designed as an elementary treatise on Surgery; and in all the editions which it has passed through, the same primary object has never been departed from." The wish of the author in its preparation has been to offer such views of scientific and practical surgery as the student and young practitioner may refer to with advantage, and more especially to supply a text-book for the Lectures annually delivered by him to the Surgical Class of University College, London. That the object of the able author has been fully attained is manifest from the number of editions throughwhich the work has passed. For a treatise on any branch of medical science to go through *seven* editions in Europe, and *four* in this country, it must possess more than ordinary merit.

The Editor informs us that he has "found occasion to add a few notes, in order to explain some views, and to introduce some new subjects: these are included in brackets." These additions are neither numerous nor extensive—indeed we cannot but regret that they are not more so, as several years have elapsed since the edition now reprinted came from the author's hands, and during that time it is known that very extensive improvements have occurred, more especially in Operative Surgery, and not a few of these belong to our own country. We presume, however, that Professor Parker abstained from a task, which must otherwise have afforded him pleasure, that the volumes might not be swelled beyond the limits prescribed by the original design of affording a text-book of convenient size for students.

